# Factors Controlling Carbon Metabolism and Humification in Different Soil Agroecosystems

**DOI:** 10.1155/2014/416074

**Published:** 2014-12-31

**Authors:** S. Doni, C. Macci, E. Peruzzi, B. Ceccanti, G. Masciandaro

**Affiliations:** Institute of Ecosystem Studies, National Research Council (CNR), Via Moruzzi 1, 56124 Pisa, Italy

## Abstract

The aim of this study was to describe the processes that control humic carbon sequestration in soil. Three experimental sites differing in terms of management system and climate were selected: (i) Abanilla-Spain, soil treated with municipal solid wastes in Mediterranean semiarid climate; (ii) Puch-Germany, soil under intensive tillage and conventional agriculture in continental climate; and (iii) Alberese-Italy, soil under organic and conventional agriculture in Mediterranean subarid climate. The chemical-structural and biochemical soil properties at the initial sampling time and one year later were evaluated. The soils under organic (Alberese, soil cultivated with *Triticum durum* Desf.) and nonintensive management practices (Puch, soil cultivated with *Triticum aestivum* L. and *Avena sativa* L.) showed higher enzymatically active humic carbon, total organic carbon, humification index (B/E_3_s), and metabolic potential (dehydrogenase activity/water soluble carbon) if compared with conventional agriculture and plough-based tillage, respectively. In Abanilla, the application of municipal solid wastes stimulated the specific *β*-glucosidase activity (extracellular *β*-glucosidase activity/extractable humic carbon) and promoted the increase of humic substances with respect to untreated soil. The evolution of the chemical and biochemical status of the soils along a climatic gradient suggested that the adoption of certain management practices could be very promising in increasing SOC sequestration potential.

## 1. Introduction

Soil systems are exposed to a variety of environmental stresses, of a natural and anthropogenic origin, which can potentially affect soil functioning. For this reason, there is growing recognition for the need to develop sensitive indicators of soil quality that reflect the effects of land management on soil and assist land managers in promoting long-term sustainability of terrestrial ecosystems [1, 2]. Soil organic matter, (SOM) providing energy, substrates, and biological diversity necessary to sustain numerous soil functions, has been considered one of the most important soil properties that contributes to soil quality and fertility [3, 4].

The SOM consists of chemical components differing in biological degradability: (i) rapid and medium turnover fractions and (ii) more recalcitrant forms that turn over slowly. The former provide immediate and short-term sources of carbon substrate for the soil biota and contribute more to nutrient cycling. The latter, on the other hand, represent long-term reservoirs of energy that serve to sustain the system in the longer term and they improve soil structure.

In order to understand the temporal dynamics of SOM in managed systems, it is therefore vital to characterize soil organic carbon quantity and quality.

In particular, by providing nutrients and physical protection for enzymes and microorganisms, soil humic carbon content has widely been recognized as an important fraction of SOM that can be used to study soil quality in ecosystems influenced by agricultural practices or adverse climate conditions. Humic substances are able to bind extracellular enzymes (humic-enzyme complexes) and preserve them from proteolysis and chemical degradation. As suggested by other studies [5, 6], the relationship between enzyme activity and humic carbon content might reflect the potential for enzyme immobilisation in soil and, therefore, the potential for soil resilience. *β*-glucosidase activity may be a particularly useful enzyme for soil quality monitoring because of its central role in SOM cycling. In particular, this enzyme, catalysing the hydrolysis of cellulose to glucose, provides an indication of the potential for soil organic matter decomposition.

Humic-*β*-glucosidase activity has been considered an important indicator of changes in soil quality resulting from environmental stress in agricultural systems [6, 7].

Agricultural management systems affect organic carbon turnover and can modify the structural composition of SOM [8].

Characterization of SOM quality in soil can be obtained by using various analytical methodologies, such as infrared, ultraviolet-visible, nuclear magnetic resonance spectroscopy, oxidative reductive polymeric degradation, and gel column filtration. Several researchers have used pyrolysis-gas chromatography (Py-GC) as a reproducible and relatively rapid technique for studying qualitative changes in the structure of SOM under different agronomic uses [9, 10]. Different peaks corresponding to the major volatile pyrolytic fragments can be used to interpret the structural evolution of SOM in terms of sources, decomposition, and stability.

Based on chemical composition, the following group of compounds can be identified: (i) aliphatics, fatty acids and sterols, (ii) carbohydrates, (iii) lignin, (iv) aromatic compounds and polycyclic aromatic hydrocarbons (PAHs), and (v) N-containing compounds.

On the other hand, other soil easily measurable descriptors can be used to study the processes related to the active labile carbon pool in soil. For instance, dehydrogenase activity, indicating the status of soil microbial activity, gives information on soil metabolism. This enzyme activity has been proposed as a valid indicator of soil quality under different agronomic practices and climatic conditions [11, 12].

Also total *β*-glucosidase activity, involved in cellulose degradation in soil, has been proposed as an early indicator of changes in organic matter status and turnover [1, 13].

Due to the complex interactions and dynamics of these soil properties, many researchers have emphasised the need to develop indices of soil quality through a combination of variables which reflect a range of soil functions, such as humification and mineralization processes, metabolism, and nutrient cycling [14].

The aim of this study was to (i) describe properties and processes that control soil organic carbon accumulation and decrease turnover rate and (ii) illustrate the importance of conservation practices and management systems that reverse the trend to degradation and facilitate carbon sequestration in soil.

These objectives may be achieved by analysing chemical, chemicophysical, and biochemical properties in order to define the most important indicators that describe organic carbon dynamics in relation to the management practices adopted in the different pedoclimatic conditions.

## 2. Materials and Methods

### 2.1. Sampling Locations

#### 2.1.1. Abanilla Experimental Site in Murcia, Spain

The site is located in Abanilla (38°12′N, 01°02′W) in open scrubland not used for agricultural purposes. The climate is Mediterranean semiarid. The mean annual rainfall is 300 mm y^−1^ and the mean annual temperature is 18°C. The studied soil is poorly developed with an ochric epipedon as the diagnostic horizon and is classified as a Haplic Calcisol (World Reference Base classification). The Abanilla site has a sandy clay loam soil (USDA classification) and it is characterized by a TOC and TIC content of 0.5% and 9%, respectively, and a pH of 6.5.

In this site, six fields of 85 m^2^ each, three treated with the organic fraction of a municipal solid waste (S-WOF treatment) and three untreated fields (S-C, control), were set up. The waste organic fraction addition was made, 16 years before soil sampling, in such a dose as to increase the SOM by 1.5%. This fraction was incorporated into the top 15 cm of the soil using a rotovator. In the S-WOF, plant cover developed spontaneously (60–70% plant coverage), while very scant vegetation grew in the control soil (20–30% plant coverage). The vegetation of the area is the typical of Mediterranean semiarid lowlands:* Pinus halepensis* Mill. and natural shrubs.

#### 2.1.2. Alberese Experimental Site in Tuscany, Italy

The site is located in Alberese (42°40′N, 11°06′E), characterized by a Mediterranean semiarid climate. The soils were taken at two agricultural areas: an organic area (I-BA) and a conventional area (I-CA). Both areas had durum wheat (*Triticum durum* Desf.) as a monoculture. In the organic area, three fields were fertilized with 100 kg ha^−1^ y^−1^ of commercial green manure, while, in the conventional area, three fields were fertilized with ammonium nitrate at a total rate of 200 kg ha^−1^ y^−1^. Organic and conventional management systems were carried out for five years. Each plot was 200 m^2^.

The Alberese site has a sandy clay loam soil (USDA classification) and it is characterized by a TOC and TIC content of 0.15% and 2.1%, respectively and a pH of 7.8. The soil is an Chromic Cambisol (World Reference Base classification). The main vegetation of the area is* Quercus ilex* L. and natural shrubs. The annual precipitation is 600 mm y^−1^ and the mean temperature is 15°C.

#### 2.1.3. Puch Experimental Site in Bavaria, Germany

The fields in Puch are located about 40 km north-west of Munich (48°10′N, 11°13′E). In this site, plant cover has been intentionally modified during the last 50 years in a long term experiment. Three plots under intensive tillage (P-IT) have been kept without plants since 1953 by ploughing twice a year and by repeated grubbing; these soils are not fertilized and are ploughed whenever vegetation appears. As a result, there is no input from plants and the SOM is constantly exposed to aeration. Three plots under conventional agriculture (P-CA) were cultivated with wheat (*Triticum aestivum* L.) and oats (*Avena sativa* L.); these soils received regular tillage, which allows some plant cover to establish itself. Three unmanaged soils were used as control soil (P-C); the control soil was abandoned and covered by low density of spontaneous vegetation. Each treated plot and each untreated (control) plot are about 200 m^2^ (total area per treatment 600 m^2^) with a sandy loam texture (USDA classification) and it is characterized by a TOC and TIC content of 1.1% and 0.03%, respectively, and a pH of 6.6. The soil is an Haplic Luvisol (World Reference Base classification), the vegetation of the area is the typical of Continental climate with a predominance of* Picea abies* (L.) H.Karst. and* Abies alba*. Mill. The annual precipitation is 900 mm y^−1^ and the mean temperature is 8°C.

The monitoring of each soil ecosystem consisted in samplings carried out once a year. In this paper, the results of the initial sampling (T1) and one year later (T2) are reported. The T1 and T2 sampling were done at the same time for the different experimental sites, even if the treatments started in different periods for the different sites.

Each soil sample was a composite of nine bulk soil subsamples randomly collected from the top layer (15 cm; 150 cm^3^ soil cores) of an homogenous area. Three composite soil samples per each replicate treatment were taken, air-dried, sieved (<2 mm), and stored at room temperature prior determining chemical, physical, and biochemical properties.

### 2.2. Methods

#### 2.2.1. Chemical, Biological, and Physical Analysis

Total organic carbon (TOC) and the total inorganic carbon (TIC) contents were measured with a LECO, U.S.A. RC-412 Multiphase Carbon/Hydrogen/Moisture Determinator. Total Nitrogen (TN) content was determined by a LECO, U.S.A. FP-528 Protein/Nitrogen Determinator. Water Soluble Carbon (WSC) was extracted using the method reported by [15]. Sodium pyrophosphate (0.1 M, pH 7.1) at 37°C for 24 h under shaking at 200 oscillation min^−1^ was used to extract Total Humic Carbon (THC) [8]. The extract was filtered on a 0.22 *μ*m Millipore membrane and passed through an ultrafiltration AMICON PM10 cut-off membrane to obtain the enzymatically active fraction >10^4^ Da (active humic carbon, AHC). The C content of WSC, THC, and AHC was determined by dichromate oxidation [16].

Total (TG) and extracellular (EG) *β*-glucosidase activities were determined on whole soil [17] and soil pyrophosphate extract fraction >10^4^ Da [8], respectively, using 0.05 M disodium (4-nitrophenyl) phosphate hexahydrate (PNG) as substrate. The 4-nitrophenol (PNP) produced by hydrolysis was extracted and determined spectrophotometrically at 398 nm [18]. Dehydrogenase activity was determined by the method of Masciandaro et al. [19], using 3-(4-iodophenyl)-2-(4-nitrophenyl)-5-phenyl-2*H*-tetrazol-3-ium chloride (INT) as electron acceptor and detecting spectrophotometrically the 1-(4-iodophenyl)-5-(4-nitrophenyl)-3-phenylformazan at 490 nm. Total porosity was determined by the method by Lowell and Shields [20].

#### 2.2.2. Chemicostructural Analyses (Pyrolysis-Gas Chromatography)

The Py-GC is based on a rapid decomposition of organic matter under a controlled high flash of temperature, in an inert atmosphere of gaseous N_2_ carrier. A gas chromatograph is used for the separation and quantification of pyrolytic fragments. Fifty micrograms of an air-dried and ground (<100 mesh) soil sample and 300 *μ*L of sodium pyrophosphate (0.1 M, pH 7.1) extract were introduced into pyrolysis quartz microtubes in a CDS Pyroprobe 190. The analysis of sodium pyrophosphate extracts gives more reliable information on native SOM.

Pyrolysis was carried out at 800°C for 10 s, with a heating rate of 10°C ms^−1^ (nominal conditions). The probe was coupled directly to a Carlo Erba 6000 gas chromatograph with a flame ionization detector (FID). Chromatographic conditions were as follows: a 3 m × 6 mm, 80–100 mesh, SA 1422 (Supelco Inc.) poropak Q packed column; the temperature program was 60°C, increasing to 240°C by 8°C min^−1^. Pyrograms were interpreted by quantification of seven peaks corresponding to the major volatile pyrolytic fragments [21]: acetonitrile (E_1_), acetic acid (K), benzene (B), pyrrole (O), toluene (E_3_), furfural (N), and phenol (Y). Acetonitrile (E_1_) is derived from the pyrolysis of aminoacids, proteins, and microbial cells. Furfural (N) is principally derived from cellulose and other aliphatic organic compounds. Acetic acid (K) is preferentially derived from pyrolysis of lipids, fats, waxes, cellulose and other carbohydrates. Phenol (Y) is derived from fresh or condensed (humic) lignocellulosic structures. Benzene (B) and toluene (E_3_) are basically derived from condensed aromatic structures of stable (humified) organic matter, particularly for benzene, since toluene must come from rings with aliphatic chains, albeit short. Pyrrole (O) is derived from nitrogenated compounds, such as nucleic acids, proteins, and microbial cells [22]. Peak areas were normalized, so that the area under each peak referred to the percentage of the total of the selected seven peaks (relative abundances). The alphabetic code used was conventional and has already been employed in previous papers [21, 23]. Peak purity of the selected major volatile fragments was checked by coupling the same chromatographic system to a mass detector HP 8000A in the same operative conditions confirming the reliability of the working conditions.

Some ratios between relative abundances of some of the peaks were determined [21]: (i) N/O: mineralization index. This index expresses the ratio between furfural, which is the pyrolytic product arising from polysaccharides, and pyrrole, which derives from nitrogenous compounds, humified organic matter, and microbial cells. The higher the ratio, the lower the mineralization of organic matter.

(ii) B/E_3_: humification index. The higher the ratio, the higher the humification of organic matter, because benzene is derived mostly from pyrolytic degradation of condensed aromatic structures, while toluene comes from aromatic uncondensed rings with aliphatic chains [21].

### 2.3. Statistical Analyses

The Statistica 7.0 software (StatSoft Inc., Tulsa, Oklahoma, USA) was used for the statistical analysis. All results are the means of three field replicates. Differences among treatments within each site were tested by analysis of variance (one way ANOVA). The means were compared by using least significant differences calculated at *P* < 0.05 (Fisher's test). In addition, the results were studied using principal component analysis (PCA), a multivariate statistical data analysis technique, which reduces a set of raw data to a number of principal components that retain most of the variance within the original variables, in order to identify possible patterns or clusters between objects and variables [24]. All raw data used in the PCA analysis were subjected to pretreatment in order to remove or reduce irrelevant sources of variation or “noise” which may interfere in the analysis. Firstly, the raw data were log transformed to reduce data heterogeneity; following this, the transformed data were standardized.

Finally, a correlation matrix of the data was also calculated in order to determine the relationship between the indicators. The significant levels reported (*P* < 0.05) are based on the Student's distribution.

## 3. Results and Discussion

The chemical, physical (Table 1), and biochemical (Table 2) variables showed very similar values at T1 and T2, which are the times corresponding to the two sampling periods, indicating the stability of soil characteristics in a short period of time.

Total organic carbon (TOC) was closely related to soil type and management systems. There were, in fact, significant differences (*P* < 0.05) in TOC content among the treatments in the three geographic areas (Table 1). The lowest TOC was found in the Abanilla unmanaged soil (control), being located in a predesertic dry area (Southern-Eastern Spain). This soil can be defined as a biologically “poor soil” [25, 26] having a low content of organic matter and microbial activity (Tables 1 and 2). The effect of waste organic fraction application on organic carbon content in the Abanilla soil was still clear 16 years later. Beneficial effects of applying organic materials on SOM are well known from several long-term experiments [27–29]. In particular, a higher content of water soluble carbon (S-WSC) was observed where organic amendment was added (S-WOF treatment), in contrast to the control soil (Table 1). In the S-WOF treated soil, plant cover developed spontaneously, while very scant vegetation grew in the control soil. The maintenance of a vegetation cover in the S-WOF soil probably had a positive influence on the input of WSC through root exudates and plant remains [30]. This labile organic carbon fraction, which is considered easily degradable by soil microorganisms [31], determined, as expected, the activation of the resident microbial populations. Beneficial effects of plants on microbial stimulation through organic exudates at the root-soil interface have been widely reported [32]. Soil dehydrogenase and total *β*-glucosidase activities were, in fact, significantly greater (*P* < 0.05) in the managed than in the control soil (Table 2). As usually reported, a positive correlation between dehydrogenase activity and WSC (*P* < 0.05, *r* = 0.75) [30, 33, 34] and *β*-glucosidase activity and WSC (*P* < 0.05, *r* = 0.92) [35] was observed.

The dehydrogenase activity, especially when referred to the energetic and immediately available C substrate, gives an idea of the metabolic potentiality of soil rehabilitation. This metabolic potential, calculated as the ratio between the activity of the viable microbial community (dehydrogenase activity) and the sources of energy for microorganisms (water soluble carbon concentration), was higher in the S-WOF with respect to the control soil.

Moreover, the higher total humic carbon (THC) and enzymatically-active humic carbon (AHC) observed in the S-WOF with respect to the control soil indicated the positive impact of organic matter addition on the maintenance of the stable carbon pool. The higher AHC also suggested the higher capacity of this stable humic fraction >10^4^ molecular weight to preserve the extracellular enzymes in an active form, as confirmed by the significantly (*P* < 0.01) higher specific extracellular *β*-glucosidase activity, calculated as the ratio between extracellular *β*-glucosidase activity and associated AHC; this specific activity allows evaluating the accumulation of enzymatically active humic pool. The preservation of the humic-enzyme complexes represents an important condition for soil resilience and their presence has been defined as a necessary condition to make the soil able to counteract the irreversible degradation (soil desertification) [36].

Significant differences in chemical and biochemical indicators related to the carbon cycle were also observed between organic (I-BA) and conventional (I-CA) agricultural soils in the Alberese site. The organic management stimulated soil metabolic potential, expressed by the ratio between the dehydrogenase activity and water soluble carbon [1, 37] (Table 2), and increased total organic carbon (TOC) and nitrogen contents (TN) and available forms of carbon (WSC, THC, AHC) (Table 1). However, the specific *β*-glucosidase, both total (expressed by the ratio between the total *β*-glucosidase activity and TOC) and extracellular (expressed by the ratio between the extracellular *β*-glucosidase activity and AHC), showed no significant difference (*P* > 0.05) between the organic and conventionally managed soils (Table 2). In general, the presence of abundant cereal crops causes less soil disturbance and stimulates microbial activity more than uncropped or intermittently cropped soil [38].

The Puch soils showed a decrease in the amount of TOC in the intensively tilled soil (P-IT), with respect to the control (P-C) and conventionally cropped (P-CA) soils. In particular, P-IT showed a lower content of active humic carbon fraction (AHC) and specific extracellular *β*-glucosidase activity (EG/AHC) (Table 2). Also the P-CA soil, although it presented higher values of chemical indicators, showed a reduced specific extracellular *β*-glucosidase activity (EG/AHC) with respect to the P-C. As already observed [38], these results indicated that the conversion of plough tillage (P-IT) to a no-till agricultural farming system (P-CA), involving a frequent use of cover crops in the rotation cycle along with adoption of integrated nutrient management, is a practice able to restore and maintain a substantial organic carbon pool in soils.

The carbon turnover may be assessed also through the chemicostructural composition of SOM as determined by the pyrolytic technique. The ratio between benzene (B) and toluene (E_3_) pyrolytic fragments, which has been considered as a “humification index,” and the ratio between furfural (N) and pyrrole (O), which has been interpreted as a “mineralization index” [8, 34], showed the same trend in the whole soil and soil extract (Table 3).

In the Abanilla site, B/E_3_ resulted higher in the organically treated soil (S-WOF) than in the control soil (S-C), suggesting the activation of humification by organic amendment; root exudates, as previously described for WSC, seem to be responsible for the low value of the N/O index found in the whole soil of the S-WOF treatment (Table 3).

Also in the Alberese site, the increase of B/E_3_ in the organic with respect to the conventional agriculture system confirmed the prevalence of the humification process over mineralization. According to the humification index, the furfural/pyrrole (N/O) ratio showed higher values in the I-BA-treated soil, thus indicating the presence of more evolved (less mineralizable) humic matter in both the whole soil and soil extract (Table 3).

In the Puch soils, significative differences were found (*P* < 0.05) in the humification index (B/E_3_) between the intensively tilled soil (P-IT), the control soil (P-C), and the conventional agriculture (P-CA). P-IT treatment, showing the lower values (*P* < 0.05), seems to be exposed to mineralization.

By considering the three management systems which were expected to negatively affect soil properties, that is, Abanilla control soil S-C, Alberese conventional agriculture I-CA, and Puch control soils P-C or P-IT, one can, on the basis of all the indicators measured, rank the soils in a decreasing order of degradation: Abanilla ≫ Alberese > Puch. In addition, being the climate one of the most important factors affecting SOM turnover, the soil degradation reflected the geographical distribution of the three selected sites, from driest to more humid places.

Therefore, Abanilla could be expected to show a slower metabolism than the other soils, which may be reflected in a different carbon turnover, and this was actually found. The management with ameliorating practices, instead, undoubtedly slows down, arrests, or even reverses soil degradation. In order to explain more clearly the factors (TOC, THC, AHC, TN, total and extracellular *β*-glucosidase, dehydrogenase, WSC, porosity, B/E_3_, and N/O) controlling carbon metabolism and humification process in the three ecosystems, principal component analysis (PCA) was performed.

Soil properties can be summarized in three independent PCs, which explained 83% of the total variance (Table 4). The first PC (PC1, 41% of the total variance) included TOC, AHC, THC, EG, Dh-ase, and B/E_3_. The statistically significant positive relation between AHC, THC, and TOC (indicators denoting significance on the same PC with the same sign) indicated the great influence of TOC on humic carbon evolution. In addition, the positive loading between these indicators and DH-ase suggests the presence of an active metabolism sustained by the readily decomposable SOM that promotes the synthesis of persistent* site-specific* humic substances (as part of humus-soil own organic matter). The evolution of the stable organic C fraction (AHC) is relevant since it determines the capability resistance and/or resilience of soils to degradation processes, particularly in extreme environments [36]. It is generally known that humic substances such as AHC, which are capable of binding active enzymes, may express both a biochemico-functional role, stabilizing extracellular enzymes also in extreme environmental conditions [31], and a chemicostructural role, their importance being related to mineral particle stabilization and the fact that they constitute a slow release nutrient source in soil ecosystems [39]. In view of this, a relationship between AHC and the functional-structural indicators (B/E_3_, total organic carbon and extracellular enzyme activity) could be expected. However, the statistically significant negative relation between TG and N/Os on the second PC suggested that the mineralization process is increased by the microbial activation of carbon cycle.

Although cause-effect relations are difficult to establish given the collinearity of variables, the positive loading of WSC and porosity on the third PC suggested a relationship between decomposable organic matter inputs and soil porosity improvement.

**Figure 1:**
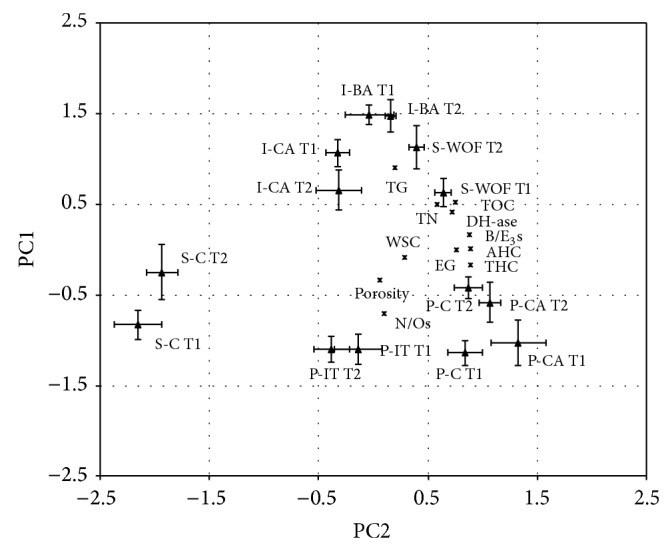
Biplot of factor scores and loadings at each sampling time (T1 and T2), in each treatment. TOC: total organic carbon; THC: total extractable carbon; AHC: extractable carbon fraction >10.000 Da; TN: total nitrogen; EG: extracellular *β*-glucosidase activity; TG: *β*-glucosidase activity; WSC: water soluble carbon; DH-ase: dehydrogenase activity; B/E_3_s: benzene/toluene whole soil; and N/Os: furfural/pyrrole whole soil.

Figure 1 provides the biplot of the PCA analysis obtained using the first two PCs. This plot gives a graphical representation of clusters of soils with similar physical-chemical and biochemical properties. The biplot indicated that organic management positively affected the soil organic carbon evolution; in fact, I-BA and S-WOF were shifted in relation to the I-CA and S-C, respectively, along positive values of PC1, which was positively associated with the indicators indicative of humification processes. These organically managed soils were also shifted along positive values of PC2, confirming their higher ability in protecting humic carbon from mineralization.

Similarly, the different Puch treatments were spread on the PC1. In particular, the P-IT treatment was shifted with respect to P-C and P-CA along negative values indicating the establishment of mineralization processes.

## 4. Conclusions

The adoption of organic (Alberese site, I-BA) and/or nonintensive management (Puch site, P-CA) practices in comparison with conventional agriculture (Alberese site, I-CA) or plough-based tillage methods (Puch site, P-IT) provoked a considerable stimulation of metabolic potential (dehydrogenase activity/water soluble carbon) and an increase of humic carbon and humic-associated enzymes.

In Abanilla site, the application of municipal solid wastes (S-WOF) stimulated the specific *β*-glucosidase activity (extracellular *β*-glucosidase activity/extractable humic carbon) with respect to untreated soil and promoted the stabilization of SOM, as showed by the increase of humic substances.

The PCA analysis was able to assess the evolution of the carbon cycle and the shift of metabolic processes towards humification or mineralization pathways in the different soil ecosystems.

The AHC showed a positive dependence on TOC and microbial activity, indicating an active metabolism sustained by the decomposable SOM, which promoted the synthesis of persistent* site-specific* humic substances. On the other hand, the negative relation between N/O index and TG indicated that the microbial activation of the carbon cycle regulates the decomposition of SOM. These results, marking the biochemical evolution and chemical status of the soils, are particularly important because they suggest that the adoption of certain management practices under different climate could have a great impact in maximizing SOC sequestration.

## Figures and Tables

**Table 1:** Chemical and physical properties at T1 and T2 sampling times. For each site, different letters indicate statistically different values among the treatments (*P* < 0.05).

		TOC	THC	AHC	TN	WSC	Porosity
		g kg^−1^	g kg^−1^	g kg^−1^	g kg^−1^	g kg^−1^	mm^3^ g^−1^
T1							
Abanilla-Spain	S-C	5.1^b^	0.81^b^	0.58^b^	0.72^b^	82^a^	192^b^
	S-WOF	27.6^a^	2.82^a^	1.96^a^	3.28^a^	225^b^	203^a^
Alberese-Italy	I-CA	12.6^b^	1.35^b^	0.89^b^	1.14^b^	63^b^	131^b^
	I-BA	18.7^a^	1.93^a^	1.35^a^	1.38^a^	70^a^	147^a^
Puch-Germany	P-CA	20.0^a^	3.09^a^	1.59^a^	1.79^a^	128^a^	204^a^
	P-IT	10.3^b^	2.05^c^	1.13^c^	0.80^c^	98^b^	161^b^
	P-C	11.5^b^	2.45^b^	1.34^b^	0.98^b^	96^b^	160^b^

T2							
Abanilla-Spain	S-C	5.9^b^	0.91^b^	0.52^b^	0.75^b^	76^b^	198^b^
	S-WOF	24.0^a^	2.63^a^	1.49^a^	2.52^a^	154^a^	217^a^
Alberese-Italy	I-CA	13.1^b^	1.38^a^	0.69^b^	0.81^b^	46^b^	142^b^
	I-BA	18.2^a^	1.41^a^	0.95^a^	1.64^a^	53^a^	154^a^
Puch-Germany	P-CA	17.3^a^	2.56^a^	1.58^a^	1.53^a^	75^a^	210^a^
	P-IT	8.0^c^	2.19^c^	1.08^c^	0.57^c^	65^b^	174^b^
	P-C	11.4^b^	2.40^b^	1.51^a^	1.18^b^	58^c^	167^b^

TOC, total organic carbon; THC, total humic carbon; AHC, active humic carbon; TN, total nitrogen; WSC, water soluble carbon.

C, control; WOF, waste organic fraction added; CA, conventional agriculture; BA, organic agriculture; IT, intensive tillage. T1, initial sampling time; T2, one year later.

**Table 2:** Biochemical properties at T1 and T2 sampling times. For each site, different letters indicate statistically different values among the treatments (*P* < 0.05).

		EG	TG	DH-ase
		mg PNP kg^−1^ h^−1^	mg PNP kg^−1^ h^−1^	mg INTF kg^−1^ h^−1^
T1				
Abanilla-Spain	S-C	3.3^b^	117^a^	1.08^b^
	S-WOF	44.1^a^	405^b^	3.21^a^
Alberese-Italy	I-CA	6.7^a^	511^b^	2.10^b^
	I-BA	6.6^a^	883^a^	2.52^a^
Puch-Germany	P-CA	16.1^b^	95^b^	4.67^a^
	P-IT	4.4^c^	82^c^	1.38^c^
	P-C	44.7^a^	182^a^	2.26^b^

T2				
Abanilla-Spain	S-C	3.6^b^	75^b^	1.78^b^
	S-WOF	31.3^a^	438^a^	5.06^a^
Alberese-Italy	I-CA	6.3^a^	639^b^	2.98^b^
	I-BA	6.4^a^	823^a^	3.58^a^
Puch-Germany	P-CA	15.8^b^	145^b^	4.31^a^
	P-IT	4.3^c^	50^c^	1.22^c^
	P-C	40.4^a^	190^a^	2.86^b^

EG, extracellular *β*-glucosidase activity; TG, total *β*-glucosidase activity; DH-ase, dehydrogenase activity.

C, control; WOF, waste organic fraction addition; CA, conventional agriculture; BA, organic agriculture; IT, intensive tillage. T1, initial sampling time; T2, one year later.

**Table 3:** Pyrolytic indices of mineralization (N/O) and humification (B/E_3_). For each site, different letters indicate statistically different values among the treatments (*P* < 0.05).

		Whole soil		AHC extract	
		N/O	B/E_3_	N/O	B/E_3_
Abanilla-Spain	S-C	1.49^a^	0.547^b^	1.21^a^	0.581^b^
	S-WOF	1.23^b^	0.827^a^	1.16^a^	0.732^a^
Alberese-Italy	I-CA	0.85^b^	0.645^b^	0.93^b^	0.796^b^
	I-BA	0.96^a^	0.738^a^	1.13^a^	0.977^a^
Puch-Germany	P-CA	1.39^a^	0.930^a^	1.28^a^	0973^a^
	P-IT	1.04^c^	0.768^b^	1.13^b^	0.910^b^
	P-C	1.25^b^	0.765^b^	1.31^a^	0.911^b^

N/O, furfural/pyrrole; B/E_3_, benzene/toluene; AHC, active humic carbon.

C, control; WOF, waste organic fraction addition; CA, conventional agriculture; BA, organic agriculture; IT, intensive tillage. Data reported as mean values of T1 (initial sampling time) and T2 (one year later), coefficient of variation of the two sampling times ranging from 2 to 10%.

**Table 4:** Principal components (PC) and component loadings related to physical, chemical, and biochemical properties determined in the different soils.

	PC1	PC2	PC3
TOC	0.745^*^	0.518	0.355
THC	0.892^*^	−0.175	0.184
AHC	0.892^*^	0.007	0.281
TN	0.585	0.496	0.611
EG	0.762^*^	−0.005	0.402
TG	0.196	0.910^*^	−0.111
WSC	0.288	−0.091	0.877^*^
DH-ase	0.722^*^	0.419	0.261
B/E_3_s	0.879^*^	0.164	−0.169
N/Os	0.101	−0.710^*^	0.233
porosity	0.057	−0.331	0.822^*^
Var. Sp.	4.498	2.196	2.382
Prp. Tot.	0.409	0.200	0.217

TOC: total organic carbon; THC: total humic carbon; AHC: active humic carbon; TN: total nitrogen; EG: extracellular *β*-glucosidase activity; TG: *β*-glucosidase activity; WSC: water soluble carbon; DH-ase: dehydrogenase activity; B/E_3_s: benzene/toluene whole soil; N/Os: furfural/pyrrole whole soil. Var. Sp.: explained variance; Prp. Tot.: total proportionality.

^*^Variables with component loadings used to interpret the PCs; threshold level: 0.7.
